# An Analysis of the Predictors of Major Bleeding After Transcatheter Aortic Valve Transplantation Using the National Inpatient Sample (2015–2018)

**DOI:** 10.7759/cureus.16022

**Published:** 2021-06-29

**Authors:** Henna Khan, Asma Gilani, Ihtisham Qayum, Taif Khattak, Furqan Haq, Muhammad Zahid Anwar, Muhammad Atif Khan, Sayyed Jalawan Asjad, Sakina Abbas, Arslan Inayat

**Affiliations:** 1 Medicine, Khyber Girls Medical College, Peshawar, PAK; 2 Internal Medicine, Khyber Teaching Hospital Peshawar, Peshawar, PAK; 3 Emergency Medicine, Hamad General Hospital, Doha, QAT; 4 Medicine, Khyber Medical College, Peshawar, PAK; 5 Internal Medicine, Texas Tech University Health Sciences Center, Amarillo, USA; 6 Internal Medicine, Royal Preston Hospital, Fulwood, GBR; 7 Medicine, Dow University of Health Sciences, Karachi, PAK; 8 Internal Medicine, University at Buffalo, Catholic Health System, Buffalo, USA

**Keywords:** transcatheter aortic valve implantation, transcatheter aortic valve replacement, tavr, tavi, aortic stenosis, major bleeding

## Abstract

Background

Transcatheter aortic valve replacement (TAVR) is now a common procedure to treat and improve quality of life, clinical outcomes, and self-sufficiency in high-risk patients with aortic stenosis, and its use has been expanding rapidly in younger and low-risk populations. The aim of this study was to evaluate the outcomes, trends, and predictors of major bleeding in patients undergoing TAVR.

Methodology

We utilized the National Inpatient Sample (NIS) data from the year 2015 to 2018. International Classification of Disease 10 codes were utilized to extract data. Baseline characteristics were compared using Pearson’s chi-square test for categorical variables and independent samples t-test for continuous variables. A multivariable logistic regression model was used to evaluate the predictors of major bleeding. Propensity matching was done for adjusted analysis to compare outcomes in TAVR with and without major bleeding. The outcomes of interest in this study were (1) predictors of major bleeding after TAVR; (2) in-hospital mortality; and (3) resource utilization in terms of cost and length of stay.

Results

A total of 34,752 weighted hospitalizations for TAVR were included in the analysis. Of the patients undergoing the procedure, 2,294 (6.6%) had a major bleed while 32,458 (93.3%) did not. At baseline, patients with coagulopathy (odds ratio [OR]: 2.03; 95% confidence interval [CI]: 1.82-2.27), congestive heart failure (OR: 1.26; 95% CI: 1.13-1.40), chronic obstructive pulmonary disease (OR: 1.41; 95% CI: 1.29-1.55), liver disease (OR: 1.96; 95% CI: 1.61-2.39), peripheral vascular disease (OR: 1.29; 95% CI: 1.17-1.43), cerebrovascular disease (OR: 1.22; 95% CI: 1.07-1.38), end-stage renal disease (ESRD) (OR: 2.17; 95% CI: 1.82-2.59), and coronary artery disease (OR: 1.17; 95% Cl: 1.06-1.30) had higher adjusted rates of odds of major bleeding. Patients who had major bleeding had a higher median cost of stay (US$60,326 vs. US$45490) and length of stay (seven vs. three days).

Conclusions

Mortality is higher in patients with major bleeding, and at baseline, coagulopathy and ESRD are significant predictors of a major bleed in patients undergoing TAVR.

## Introduction

In the medium-to-high-risk elderly patients who suffer from severe aortic stenosis and calcified aortic valve disease, transcatheter aortic valve replacement (TAVR) has proven to be an effective and minimally invasive procedure [1,2]. The three landmark PARTNER trials have exhaustively evaluated the efficacy and side effect profile of TAVR which has stood the test of time [2,3]. It is an effective alternative to surgical valve replacement and has been widely adopted in the United States [3,4].

According to the Society of Thoracic Surgeons/American College of Cardiology Transcatheter Valve Therapy Registry investigators, TAVR has been reported to have a 92% procedural success rate [5]. In patients with aortic stenosis, TAVR has led to constant improvement in clinical outcomes along with improved techniques as the procedure has undergone significant evolution given increased operator experience [6,7].

Major bleeding is a life-threatening complication of surgical aortic valve replacement (10% surgical aortic valve replacement [SAVR] versus 6% for TAVR) [1]. However, it is important to mention that despite complication rates being lower with TAVR compared to the surgical option, major bleeding remains a significant complication identified in the landmark PARTNER trials [1-3].

One of the most frequent complications post-TAVR with the first PARTNER trial was major bleeding which reported 10.2% bleeding events [8]. Major bleed is known to be associated with significant mortality and morbidity even though it is less frequent compared to SAVR [9,10]. Post-TAVR, major bleeding was reported to increase one-month mortality by 32% [11]. Data on outcomes of TAVR patients who develop major bleeding remain limited. Hence, the main objective of this study was to comprehensively evaluate the predictors and outcomes of major bleeding in TAVR using the National Inpatient Sample (NIS) database.

## Materials and methods

The NIS database was used to identify cases of TAVR performed during 2015-2018. NIS is a publicly available database and does not contain any patient-sensitive information. Hence, this study did not require ethical board approval or informed consent [12]. NIS data are collected annually and are representative of 20% of the hospitalizations in the United States. The cost of hospitalization is also recorded in US dollars which comprises the cost incurred in return for services provided.

National analysis was performed using the international classification of diseases (ICD)-9 (3505, 3506), and ICD-10 (02RF3) codes were utilized to identify all cases of TAVR. We extracted cases from all available procedure fields. Patients younger than 18 years of age were not included in the analysis. Data on comorbidities are provided by the NIS. Major bleeding in our analysis was defined as any bleeding requiring transfusion. The outcomes of interest for our study were (1) predictors of major bleeding after TAVR; (2) in-hospital mortality; and (3) resource utilization in terms of cost and length of stay.

We used the discharge weights provided by NIS to perform analysis on weighted hospitalizations. We used the Mann-Whitney U test for all continuous variables as they are not normally distributed, and the chi-square test was used for categorical variables. To test for the non-normality of data we used the Shapiro-Wilk test. Entry method was used to develop a binary logistic model including baseline comorbidities such as obesity, weight loss, metastatic cancer, lymphoma, solid organ tumor, alcohol use, coagulopathy, hypothyroidism, chronic obstructive pulmonary disease (COPD), cerebrovascular disease (CVA), congestive heart failure (CHF), coronary artery disease (CAD), diabetes mellitus, hypertension, liver disease, chronic kidney disease (CKD), and peripheral vascular disease (PVD). Demographic factors such as age, sex, race, median income, and hospital location were also included in the analysis. Observations with less than 11 cases were not reported in compliance with the Health Cost and Utilization Project. R software version 3.5 was used for all analyses. We considered a p-value of <0.05 to be statistically significant.

## Results

**Figure 1:**
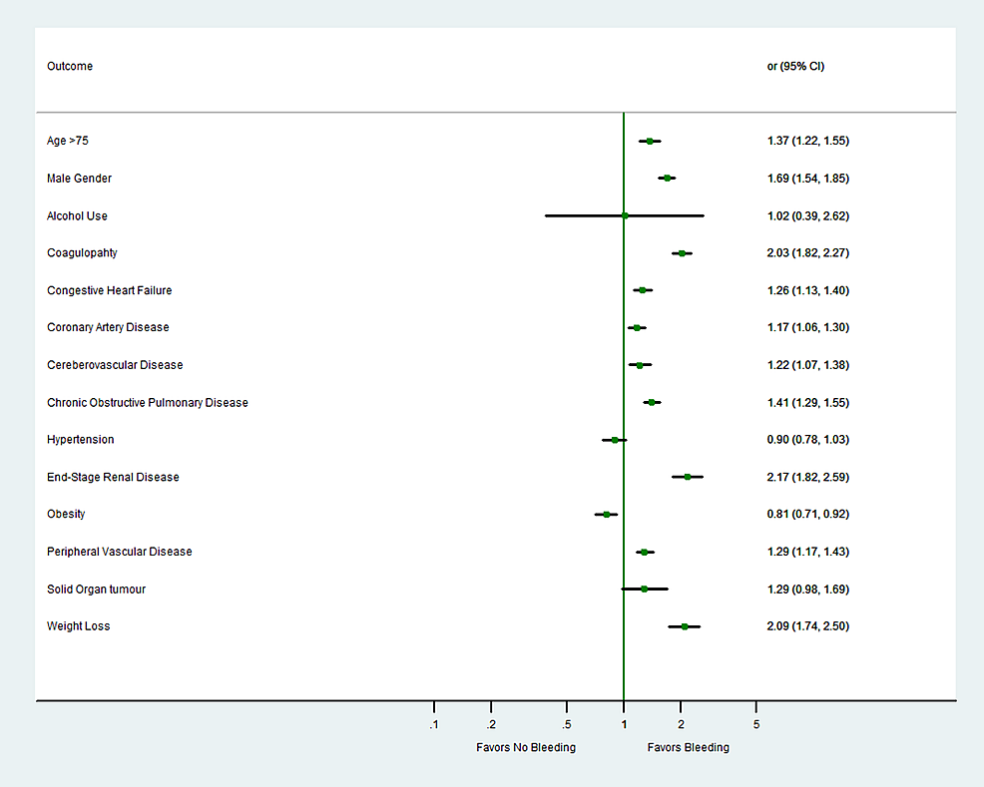
Adjusted odds of predictors of in-hospital major bleeding in patients undergoing TAVR. TAVR: transcatheter aortic valve replacement

A total of 34,752 weighted hospitalizations for TAVR were included in the analysis. Of the patients undergoing the procedure, 2,294 (6.6%) had bleeding complications while 32,458 (93.3%) did not. The detailed baseline characteristics are summarized in Table 1. At baseline, patients with coagulopathy (odds ratio [OR]: 2.03; 95% confidence interval [CI]: 1.82-2.27), CHF (OR: 1.26; 95% CI: 1.13-1.40), COPD (OR: 1.41; 95% CI: 1.29-1.55), liver disease (OR: 1.96; 95% CI: 1.61-2.39), PVD (OR: 1.29; 95% CI: 1.17-1.43), CVA (OR: 1.22; 95% CI: 1.07-1.38), end-stage renal disease (ESRD) (OR: 2.17; 95% CI: 1.82-2.59), and CAD (OR: 1.17; 95% Cl: 1.06-1.30) had higher adjusted rates of odds of major bleeding (Table 1; Figure 1). Patients who had major bleeding had a higher median cost of stay (US$60,326 vs. US$45,490) and length of stay (seven vs. three days) (Table 2).

**Table 1:** Baseline characteristics and predictors of major bleeding in patients after TAVR. OR: odds ratio; CI: confidence interval; IQR: interquartile range; CHF: congestive heart failure; CAD: coronary artery disease; COPD: chronic obstructive pulmonary disease; ESRD: end-stage renal disease; PVD: peripheral vascular disease; TAVR: transcatheter aortic valve replacement

			Multivariate analysis OR (95% CI)
Variable. (%)	Without major bleed (32,458)	With major bleed (2,294)	No bleeding vs. bleeding
Age, median (IQR)	81 (75-86)	82 (76-87)	1.37 (1.22-1.53)
Female gender	14,823 (45.7%)	1,290 (56.2%)	1.69 (1.54-1.85)
Caucasian	27,107 (87.2%)	1,828 (82.6%)	Reference
African Americans	1,267 (4.1%)	146 (6.6%)	1.53 (1.27-1.86)
Hispanics	1,480 (4.8%)	148 (6.7%)	1.48 (1.24-1.78)
Alcohol use	52 (0.2%)	5 (0.2%)	1.02 (0.39-2.62)
Hypothyroidism	6,591 (20.3%)	489 (21.3%)	0.96 (0.86-1.07)
Coagulopathy	3,792 (11.7%)	525 (22.9%)	2.03 (1.82-2.27)
CHF	23,725 (73.1%)	1,793 (78.2%)	1.26 (1.13-1.40)
CAD	22,458 (69.2%)	1,627 (70.9%)	1.17 (1.06-1.30)
Cerebrovascular disease	3,711 (11.4%)	339 (14.8%)	1.22 (1.07-1.38)
COPD	9,785 (30.1%)	863 (37.6%)	1.41 (1.29-1.55)
Diabetes mellitus	5,144 (15.8%)	282 (12.3%)	0.85 (0.75-0.98)
Hypertension	28,886 (89.0%)	2,038 (88.8%)	0.90 (0.78-1.03)
Liver disease	949 (2.9%)	144 (6.3%)	1.96 (1.61-2.39)
ESRD	1,128 (3.5%)	186 (8.1%)	2.17 (1.82-2.59)
Obesity	5,545 (17.1%)	296 (12.9%)	0.81 (0.71-0.92)
PVD	7,000 (21.6%)	629 (27.4%)	1.29 (1.17-1.43)
Weight loss	979 (3.0%)	168 (7.3%)	2.09 (1.74-2.50)
Metastatic cancer	210 (0.6%)	19 (0.8%)	1.20 (0.71-2.03)
Lymphoma	221 (0.7%)	16 (0.7%)	1.21 (0.72-2.03)
Solid organ tumor	780 (2.4%)	72 (3.1%)	1.29 (0.98-1.69)
Income 0-25^th^ percentile	6,838 (21.4%)	481 (21.3%)	Reference
25^th^–50^th^	8,255 (25.8%)	493 (21.8%)	0.92 (0.80-1.05)
50^th^–75^th^	8,544 (26.7%)	608 (26.9%)	1.11 (0.98-1.27)
75^th^–100^th^	8,336 (26.1%)	677 (30.0%)	1.26 (1.11-1.44)
Urban	277 (0.9%)	21 (0.9%)	Reference
Urban nonteaching	2,997 (9.2%)	238 (10.4%)	1.09 (0.66-1.81)
Urban teaching	29,184 (89.9%)	2,035 (88.7%)	0.91 (0.56-1.48)

**Table 2:** In-hospital outcomes of patients with and without bleed in TAVR. TAVR: transcatheter aortic valve replacement

Outcome	No bleed	Bleed	P-value
Died during hospitalization	432 (1.3%)	146 (6.4%)	<0.01
Median length of stay (days)	3 (2-5)	7 (4-13)	<0.01
Median cost of stay (US$)	45,490 (35,640-57,665)	60,326 (45,756-81,222)	<0.01

## Discussion

Approximately 6% of patients who undergo TAVR have major bleeding. Of the patients who have major bleeding, 6.4% die during hospitalization. We also identified important baseline characteristics such as age greater than 75, female sex, and history of ESRD, liver disease, PVD, CHF, and CAD.

Major bleeding or vascular complications are expected to decrease as TAVR technology evolves into smaller device sizes [13,14]. However, bleeding complications have been underreported and are inconsistent in the early literature [10]. Patients who undergo TAVR are typically frail, elderly, and are at a risk for both bleeding and ischemic complications [12,15,16]. Careful risk and benefit evaluation is warranted to identify the antithrombotic regimen, as major late bleeding complications are not only frequent but also associated with an increased risk of total mortality [17]. With baseline hematological problems, higher bleeding complications related to coagulation factors and platelets were identified in our study. According to the literature, our findings are reinforced by a good association between blood disorders and major bleeding events [16].

According to a previous study, major bleeding is associated with a three-fold increase in one-year mortality following TAVR and SAVR [18]. A previous study reported that major bleeding and life-threatening bleeding after TAVR, as defined by the Valve Academic Research Consortium (VARC) criteria, occurred in approximately 15-20% of TAVR procedures [13]. Factors that increase the risk of bleeding include a high prevalence of CKD, peripheral vasculopathy, acquired thrombocytopenia, and acquired reversible von Willebrand factor deficiency [16,19-21]. It is important to note that our definition of major bleeding was not based on well-validated VARC-II criteria similar to previous studies, instead, we used ICD codes to define major bleeding. However, our study findings are in agreement with prior reported data as we report higher bleeding risk with PVD (1.29 times) and coagulopathy (2.03 times).

After the procedure, patients undergoing TAVR had lower rates of major or life-threatening bleeding (11.3% vs. 20.9%), acute kidney injury (AKI) stage II and III, and cardiogenic shock compared to those undergoing SAVR [13,20-26]. Due to the inherent platelet dysfunction, patients with ESRD have a propensity to bleed and are associated with high mortality [19,20]. Similarly, due to associated coagulopathy, liver disease patients have an increased tendency to bleed [27]. In a previous meta-analysis, patients with chronic liver disease had a higher incidence of bleeding complications, need for blood transfusions, and mortality, which was further exacerbated by antiplatelet drug use [28,29]. As reported by Tchetche et al., 38.9% of patients undergoing TAVR received at least one transfusion [10,13,19,29]. Our estimate of major bleeding complications in TAVR was 6.6%, which we believe to be a more contemporary estimate. Moreover, we report 2.17 and 1.96 times higher odds of bleeding with ESRD and liver disease, respectively. Interestingly, our study found that obesity and hypertension are associated with a lower risk of bleeding. The phenomenon of the obesity paradox has been seen in our study, which was previously described for diseases such as myocardial infarction, heart failure, and renal disease [30]. Similarly, prior TAVR literature data based on NIS reported hypertension as a protective factor against mortality [18].

NIS provides an opportunity to identify characteristics from a large sample size to evaluate the predictors. For instance, previously, it was found that peptic ulcer disease and colon cancer lead to high gastrointestinal bleeding after TAVR. This led to a change in the recommendation to perform a pre-TAVR colonoscopy and proton pump inhibitor use to mitigate the risk. Similarly, patients undergoing TAVR who were found to have a high risk of AKI underwent pre-TAVR intravenous hydration along with minimal contrast use during the procedure. Our study aimed to replicate this by providing data on these predictors so that mitigation measures can be taken against bleeding.

Our study findings are not without limitations. For instance, we could not extract data on antiplatelet and anticoagulant use as they are not available in the NIS database. Similarly, data on the cause of death are not available in the NIS. Moreover, NIS coding errors cannot be completely ruled out.

## Conclusions

We report that at baseline, ESRD, liver disease, PVD, CHF, CAD, age greater than 75, and female gender are associated with a higher risk of bleeding after TAVR. It is of utmost importance to identify patients who are at a high risk of developing complications as TAVR expands to a rapidly aging population.
